# Supplementary material for: Motives and Passion of Adults from Pakistan toward Physical Activity
Source: Int J Environ Res Public Health. 2022 Mar 11;19(6):3298. doi: 10.3390/ijerph19063298 (PMC8951450; doi:10.3390/ijerph19063298)
Supplement: Supplementary file 1 [file ijerph-19-03298-s001.zip › S1.pdf]

We translated the Physical Activity and Leisure Motivation Scale and Passion Scale from the source language (English) to the target language (Urdu), using back-translation based on Brislin's model [1]. Two experienced bilingual translators (fluent in both English and Urdu) independently translated the PALMS and PS from English to Urdu. Three experts in the areas of Physical Education, Sports Sciences, and Sport Psychology, compared both translations to create the Urdu versions of the PALMS and PS. Another group of two bilingual translators independently back-translated the Urdu versions to English. An independent team of professionals reviewed both back-translated versions and compared them to the original English version. They made small changes, such as to the grammar, in order to improve clarity. Then, we distributed the final versions of the PALMS and PS to experts and postgraduate students from the Department of Physical Education and Sports Sciences at a university in Pakistan. We asked the experts to assess whether the content of the questionnaires was culturally appropriate to the Pakistani population.

Validation of the Physical Activity and Leisure Motivation Scale

Psychometric Properties of the Physical Activity and Leisure Motivation Scale

The internal consistency of the eight motives in the Urdu version of PALMS was measured by calculating Cronbach's alpha coefficient (α) for each motive subscale. The α coefficients of the eight motives in the 40-item PALMS scale were all above the recommended threshold of .70 [2,3], so they all showed satisfactory internal consistency reliability (mastery, α = .90, enjoyment = .89, psychological condition = .84 physical condition = .88, appearance = .90, others' expectations = .84, affiliation = 86, and competition/ego = .87).

Descriptive statistics along with reliability and validity measures are presented in Table 1. A Composite Reliability index (CR) was calculated to further examine the reliability of the questionnaire. The CR for each factor exceeds the minimum recommended level of 0.70 [4]. The Average Variance Extracted (AVE) values for all factors are larger than the suggested level of 0.50 [5].

Test-retest Reliability

Test-retest reliability was examined with a sub-sample of the main sample, consisting of 83 respondents. In addition to the original testing of PALMS, these participants completed PALMS again four months later. PALMS showed sound test-retest reliability, based on Pearson's product moment correlations (r) between the two tests over this 4-month period, with mastery, $r = .94$, $p < .01$, enjoyment, $r = .88$, $p < .01$, psychological condition, $r = .84$, $p < .01$, physical condition, $r = .91$, $p < .01$, appearance, $r = .96$, $p < .01$, others' expectations, $r = .83$, $p < .01$, affiliation, $r = .87$, $p < .01$, and competition/ego, $r = .91$, $p < .01$. These are high values for test-retest reliability correlations over the period of four months, which is longer than is widely employed. Test-retest reliability values typically reduce as the period between test and retest increases, but the values here are high.

Table. Descriptive Statistics, Reliability, and Validity of the Factors of PALMS

Factors	No of items	M	SD	CR	AVE
Mastery	5	19.97	4.87	0.89	0.62
Enjoyment	5	17.93	5.60	0.89	0.62
Psychological Condition	5	18.84	4.41	0.84	0.51
Physical Condition	5	20.38	4.58	0.88	0.60
Appearance	5	19.47	5.33	0.90	0.65
Other's Expectations	5	14.98	5.18	0.82	0.51
Affiliation	5	16.79	5.13	0.86	0.56
Competition/ego	5	17.55	5.26	0.87	0.57

CR = Composite Reliability, AVE = Average Variance Extracted

Confirmatory Factor Analysis of the Physical Activity and Leisure Motivation Scale

To examine factorial validity of the Urdu version of PALMS, CFA was conducted through Structural Equation Modelling (SEM), using AMOS. The fit indices values showed an indication of good fit of the established model for the given data. The model fit was examined in one key step. In this step the indices of absolute and relative fit (CFI, NFI, and RMSEA) were examined. Because the chi-square test of absolute model fit is sensitive to sample size and number of parameters, investigators often turn to various descriptive fit statistics to assess the overall fit a model has to the data. Hu and Bentler [6] recommended that χ^2/df between 1 and 3, the Root Mean Square Error of Approximation (RMSEA) and standardized root mean square residual (SRMR) values .08 or less, and Comparative Fit Index (CFI) and Non-normed Fit Index (NNFI) values of .9 or higher represent good fit, while .9 ≤ .8 is considered permissible, in certain circumstances. Since RMSEA for the initial model was .07 and the CFI and NNFI values were .87 and .85 respectively, while χ^2/df was 4.07, the model did not fit well, according to the descriptive measures of fit.

The process of model modification started as recommended by the modification indices. According to Kenny [7], the modification indices proposes covariance between errors of items that are shown to match in content. Arbuckle [8] recommended the standards of modification indices of error covariance should be no less than 4.0. Therefore, only the covariance of variables was combined for which chi square change was 4 or greater (see Figure S1). After that, once again the indices of absolute and relative fit (CFI, NNFI, and RMSEA) were compared. The RMSEA and SRMR for the modified first-order model after calculation of the covariance were shown to be .05 and .04 respectively, whereas the CFI and NNFI values were revealed to be .93 and .92 respectively, while χ^2/df was 2.59. These values were strong enough to fit the first-order model as shown in Figure S1. The Standardized Regression Loadings for the eight factors of the Urdu Version of PALMS are shown in Figure S1. All the standardized loadings of items exceeded 0.50. Standardized regression loadings should be 0.50 or greater [9,10].

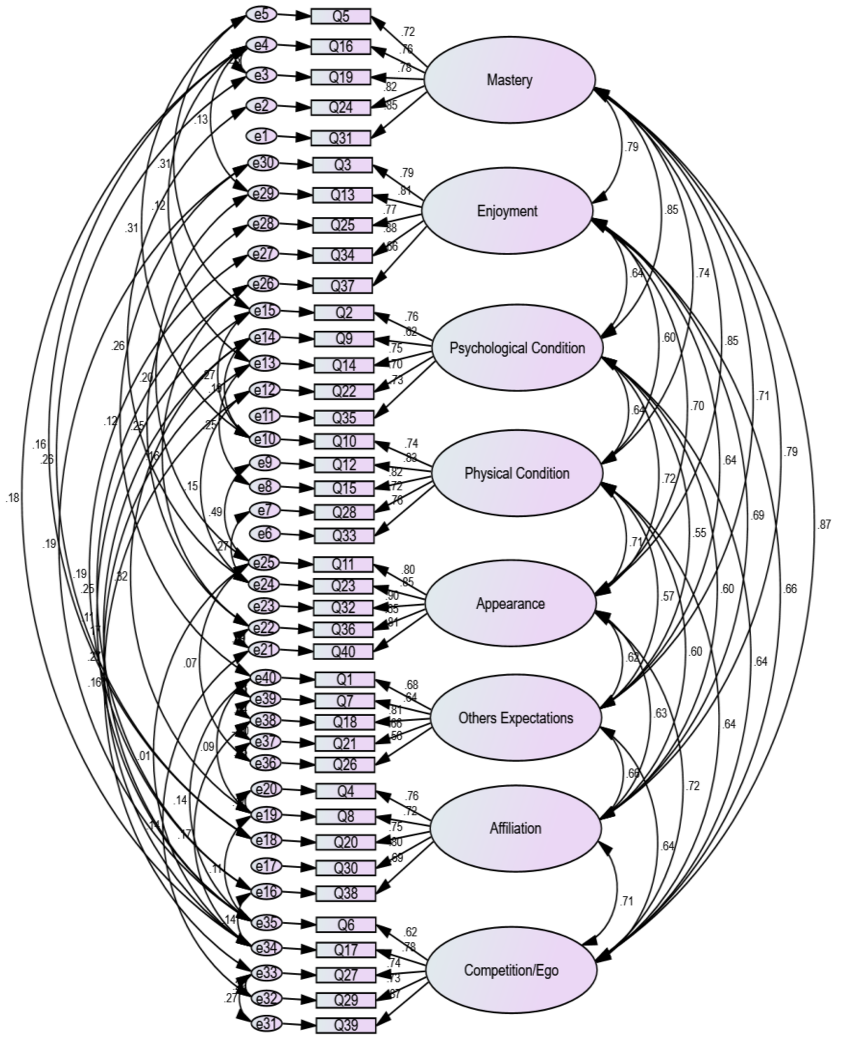

Figure S1. Standardized Regression Loadings of First Order CFA after Adding Covariance

Validation of the Passion Scale

Psychometric Properties of the Passion Scale

The internal consistency of HP and OP subscales of the Urdu version of the PS was measured by calculating α coefficients. The alpha reliability coefficient of the 6-item HP subscale was, α = .90, and the coefficient for the 6-item OP subscale was, α = .89. Thus, both HP and OP showed acceptable α coefficient values (≥ .70; [2].

A CR was calculated to determine the reliability of the PS. The CR exceeded the minimum recommended level of 0.70 [4]. The CR found for HP was, CR = 0.90, and the CR for OP was, CR = 0.89. The average variance extracted (AVE) values were larger than the suggested threshold level of 0.50 [11]. The AVE for Harmonious Passion was 0.60 and Obsessive Passion was 0.58.

Test-retest Reliability

The PS Urdu Version (PS-U) revealed good test-retest reliability, with the 83 respondent sub-sample over a 4-month period of HP, $r = .88$, $p < .01$, and OP, $r = .76$, $p < .01$.

Confirmatory Factor Analysis of the Passion Scale

To examine factorial validity of the Urdu version of the PS, CFA was conducted through SEM, using AMOS. The fit indices values were calculated to test for a good fit of the data collected from the present sample against the established 2-factor model of the PS, comprising a HP factor and an OP

factor. The model fit was examined in one key step. In this step, the indices of absolute and relative fit (CFI, NFI, and RMSEA) were compared. The RMSEA for the initial model was .12 and the CFI and NNFI values were .90 and .87 respectively, while χ^2/df was 8.97. The model did not fit well, according to the descriptive measures of fit.

The process of model modification started as recommended by the modification indices. Thus, only the covariance of variables was added for which chi square change was 4.0 or greater (see Figure S2). After that, once again the indices of absolute and relative fit (CFI, NNFI, and RMSEA) were compared. The RMSEA and SRMR for the modified first order model after calculation of the covariance were .05 and .04, respectively. The CFI and NNFI values were .99 and .98 respectively, while χ^2/df was 2.55. These values demonstrated a good fit to the first order model as shown in Figure S2. The standardized regression loadings for the two factors of the Urdu Version of PS are presented in Figure S2. Standardized regression loadings should be 0.50 or greater [9,10]. These loadings are all well above the threshold of 0.50, confirming that all six standardized regression loadings of items in the HP subscale and all six standardized regression loadings of items in the OP subscale of the PS were acceptable.

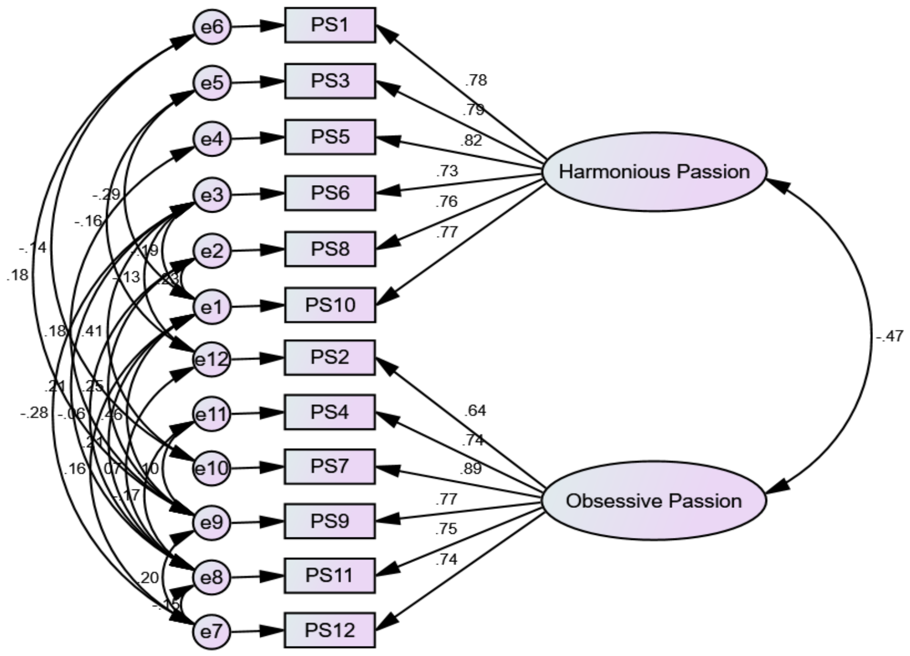

Figure S2. Standardized Regression Loadings of First Order CFA after Adding Covariance

1. Brislin, R.W. Back-Translation for Cross-Cultural Research. *J Cross Cult Psychol* **2016**, *1*, 185-216, doi:10.1177/135910457000100301.
2. Cortina, J.M. What is coefficient alpha? An examination of theory and applications. *Journal of Applied Psychology* **1993**, *78*, 98-104.
3. Cortina, J.M. What is coefficient alpha? An examination of theory and applications. *Journal of Applied Psychology* **1993**, *78*, 98-104.
4. Bagozzi, R.P.; Yi, Y. On the Evaluation of Structural Equation Models. *Journal of the Academey of Marketing Science* **1988**, *16*, 74-94, doi:10.1007/BF02723327.
5. Salama-Younes, M.; Hashim, M. Passion, vitality and life satisfaction for physically active old adults. *J Posit Psychol* **2018**, *13*, 309-319.

6. Hu, L.t.; Bentler, P.M. Cutoff criteria for fit indexes in covariance structure analysis: Conventional criteria versus new alternatives. *Struct Equ Modeling* **1999**, *6*, 1-55, doi:10.1080/10705519909540118.
7. Kenny, D.A. Correlated errors. Re-specification of latent variable model. *Retrieved from http://davidakenny.net/cm/respec.html* **2011**.
8. Arbuckle, J.L. IBM. SPSS. AMOS. *User's Guide. IBM Corp* **2012**.
9. Hair, J.F.; Black, W.C.; Babin, B.J.; Anderson, R.E. Multivariate Data Analysis: A Global Perspective. *7th Edition, Pearson Education, Upper Saddle River.* **2010**.
10. Civelek, M.E. Essentials of structural equation modeling. *Essentials of Structural Equation Modeling (2018)* **2018**.
11. Hair, J.F.; Sarstedt, M.; Ringle, C.M.; Mena, J.A. An assessment of the use of partial least squares structural equation modeling in marketing research. *J Acad Mark Sci* **2011**, *40*, 414-433, doi:10.1007/s11747-011-0261-6.
